# Long-Term Muscular Atrophy and Weakness Following Cessation of Botulinum Toxin Type A Injections in the Flexor Digitorum Muscle of Musicians with Focal Hand Dystonia

**DOI:** 10.3390/toxins15040296

**Published:** 2023-04-18

**Authors:** Christos I. Ioannou, Franziska L. Hodde-Chriske, Eckart Altenmüller

**Affiliations:** 1 CYENS—Centre of Excellence, 1016 Nicosia, Cyprus; 2 Institute of Music Physiology and Musicians’ Medicine, Hanover University of Music, Drama and Media, 30175 Hanover, Germany; fidelfranzi@gmail.com (F.L.H.-C.); eckart.altenmueller@hmtm-hannover.de (E.A.); 3 Hanover Medical School, 30625 Hanover, Germany

**Keywords:** ultrasound, strength, muscle thickness, botulinum toxin, side effects, symmetry index

## Abstract

The present study assessed muscular atrophy and weakness of the flexor digitorum superficialis (FDS) and profundus (FDP) muscle as possible long-term side effects of botulinum toxin (BoNT) injections in hand dystonia patients after the termination of their treatment. For the assessment of both parameters, a group of 12 musicians diagnosed with focal hand dystonia was compared with a group of 12 healthy matched musicians. The minimum and maximum times since the last injection across patients were 0.5 to 3.5 years, respectively. The thickness and strength of the FDS and FDP were assessed via ultrasonography and a strength measurement device. Group differences were estimated through the calculation of the symmetry index between the dominant and non-dominant hand. The results revealed that compared to the control group, thickness and flexion strength of the injected FDS and FDP were decreased by 10.6% ± 5.3% (95% CI) and 12.5% ± 6.4% (95% CI), respectively, in the patient group. The amount of weakness and atrophy was predicted significantly by the total amount of BoNT injected throughout the entire treatment period. In contrast, the time after the last injection did not predict the amount of strength and muscle mass recovery after the cessation of the treatment. The current study revealed that even up to 3.5 years after the termination of BoNT injections, long-term side effects such as weakness and atrophy can still be observed. We suggest that the total BoNT dose should remain as small as possible to reduce long-lasting side effects to the minimum. Although side effects differ significantly among patients, a potential full recovery of atrophy and weakness after the cessation of BoNT treatment might be observed after periods longer than 3.5 years.

## 1. Introduction

Focal hand dystonia in musicians is a rare, but with respect to professional activities, frequently devastating neurological movement disorder. It causes uncontrollable muscular contractions of the wrist and finger flexor and extensor muscles and a degradation of coordination of the affected finger movements [1]. Not only can musicians be affected by focal hand dystonia, but so can other professionals such as typists or athletes [2,3,4]. One of the many approaches to alleviate symptoms is the injection of affected muscles with Botulinum Toxin (BoNT) Type A [5]. The injection causes a chemical denervation resulting in a loss of muscular contractility [6] and thereby reducing dystonic cramping. Since the affected musicians have high demands concerning their muscular functionality to be able to play their musical instruments professionally, BoNT injection therapy often has a narrow therapeutic window, representing a special challenge to balancing symptomatic relief with the functional integrity of the musculoskeletal system [7]. However, the symptomatic effect of a BoNT injection wears off and re-injections become necessary at a recommended interval of 3–4 months [8,9].

In several clinical studies, BoNT was rated as a safe and efficient treatment option, even with regard to the long-term outcome (for a review, see [10,11,12]). However, single or multiple injections of BoNT may also lead to the development of short- and/or long-term effects such as muscular weakness and atrophy. These effects can of course be considered functionally therapeutic as they can lead to symptomatic relief, and the patient’s satisfaction should always be a priority. However, the injections of BoNT may also lead to irreversible changes in neuro-muscular functions, especially in cases of repeated injections. Studies of long-term outcomes on human subjects remain rare and methodologically limited. For instance, in a study by Cole et al. [13], ten patients with focal hand dystonia were examined in a double-blind placebo-controlled trial. Three months after the injection, treatment satisfaction was much better in the BoNT group while strength, assessed subjectively with the MRC (Medical Research Council [14]) scale, was not much lower than in the placebo group. However, all patients had received BoNT injections regularly before the experiment with just a three-month-long break prior to the experiment. For this reason, the reported MRC values of this trial should be interpreted with caution. Another study focusing on the long-term effects of BoNT in patients with focal hand dystonia was conducted by Lungu et al. [15]. Patients were examined in a ten-year follow-up study using a subjective four-point self-rating scale assessing effectiveness and the MRC scale assessing weakness. Patient satisfaction in this examination was high, while only minor weakness was reported. In contrast, when muscular atrophy/mass was assessed with objective methods (MRI and muscle biopsies), a neurogenic atrophy was still observed even 12 months after a single injection of BoNT Type A [16]. However, in this study, only two healthy volunteers were tested.

The side effects of muscular weakness and atrophy have been studied with greater sample sizes and objective measurement methods in animal model studies. An immediate effect of BoNT injections was shown using EMG in a rabbit model by Park et al. [17]. EMG muscle activity was significantly decreased within the first eight hours after an injection of 20 units of BoNT in the masseter muscle of New Zealand White rabbits. The recovery of muscle strength and mass was investigated by Fortuna et al. [18]. The study indicated an incomplete recovery of strength and muscle mass in injected and even non-injected muscles in New Zealand White rabbits with a loss of contractile material still present after a recovery period of six months. In another rabbit model study, an increasing loss of muscle mass and contractile material was observed with increasing monthly injections from one to three and six months, respectively [19]. The contractile material was replaced with fatty tissue resulting in a lower muscle quality. The development of weakness and atrophy was also examined using Sprague-Dawley rats. A loss of muscle mass could be observed 128 days (=18.3 weeks) after a single injection of BoNT [20] with a clear dose dependency of the amount of atrophy and weakness. The onset of recovery itself was found to be dose-dependent, with Billante et al. [21] revealing that higher BoNT doses resulted in a later onset of recovery and a higher chance for the development of muscular atrophy. While rats in the low-dose BoNT group reached their baseline EMG values again after 55–75 days (=7.9–10.7 weeks), rats in the high-dose group (10 units) never exceeded 35% recovery after an observation period of 22 weeks. In a rat model study by Ma et al. [22], muscle mass and strength were significantly reduced 1–2 weeks after BoNT injection but returned to nearly normal values after a period of six months. However, neuromuscular changes were still present up to one year later.

Sometimes, patients suffering from focal hand dystonia who responded to BoNT injections decide to discontinue their treatment even after years of injection therapy. The reasons vary and may include a change of provider, the short duration of effect, symptom improvement through other treatment options, and many more. For dystonic musicians in particular, an adaptation of their repertoire, the shift from a solo to a teaching career, the high treatment cost, the alternative available treatment options such as sensorimotor retraining, and an insufficient response/benefit of the injection therapy are reasons why they decide to terminate their BoNT treatment [15,23]. Therefore, besides adding to the limited findings on human subjects, the current study attempted to objectively assess muscular atrophy and weakness via ultrasonography and finger strength assessments [24] in dystonic musicians who have terminated their BoNT treatment therapy. The main research question of the current investigation was the following: to what degree do atrophy and weakness persist/recover over time in focal hand dystonia patients after the cessation of a multiple BoNT injection therapy?

## 2. Results

**Figure 1:**
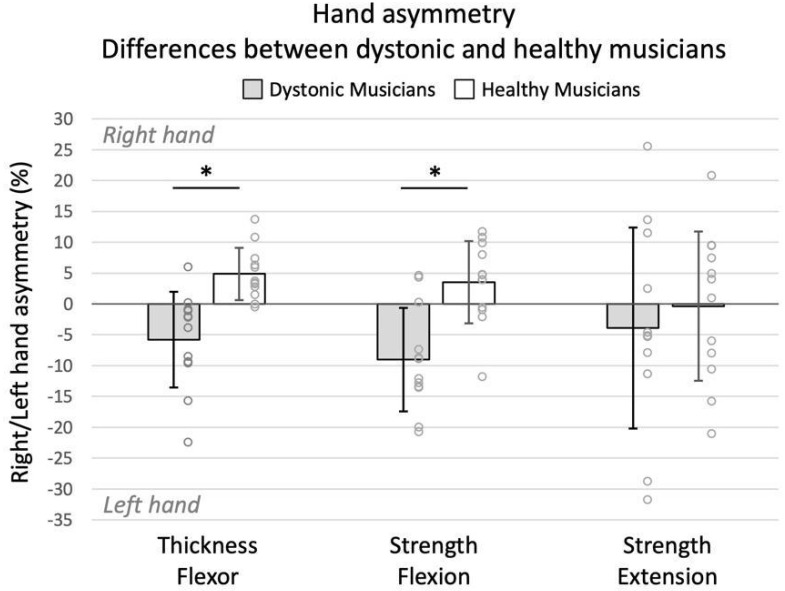
Asymmetry towards right (dominant) or left (non-dominant) hand between dystonic and healthy musicians. Positive and negative values indicate larger asymmetry towards the right and left hand, respectively, whereas zero indicates no asymmetry between the two hands. Asterisk indicates a significant difference at the Bonferroni-adjusted *p* value (<0.017). Error bars indicate standard deviation.

Compared to the healthy controls (n = 12), dystonic musicians (n = 12) who had terminated their BoNT treatment showed a significantly larger asymmetry toward the left (non-injected) hand for flexor thickness (*t*(22) = −4.171, *p* < 0.001, *r* = 0.66) and flexion strength (*t*(22) = −4.058, *p* < 0.001, *r* = 0.65). Extension strength asymmetry indicated no differences between the two groups (*t*(22) = −0.607, *p* > 0.05, *r* = 0.13) (Figure 1). Significance was accepted at *p* < 0.017 (Bonferroni).

**Figure 2:**
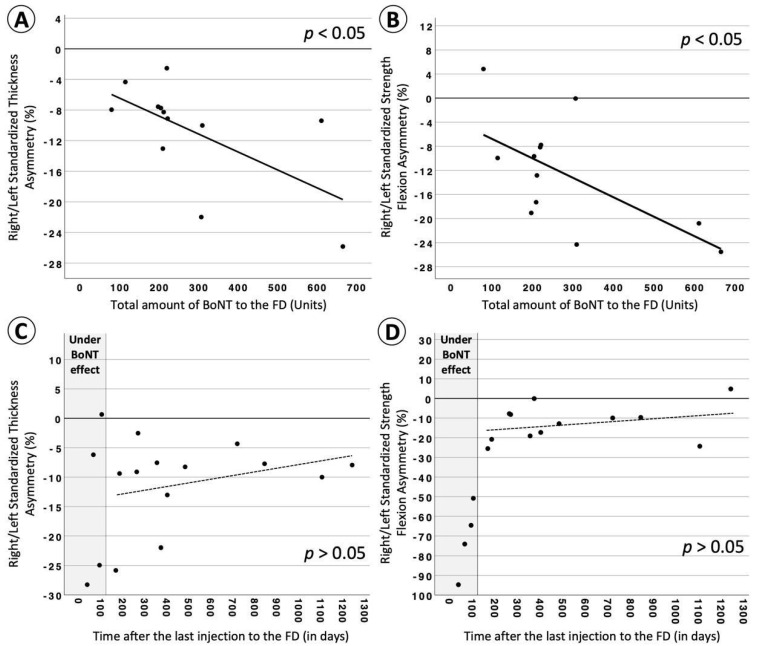
The “Total amount of BoNT to the FD” significantly predicted the degree of strength (**A**) and thickness (**B**) standardized asymmetry in dystonic musicians who terminated their treatment. “Time after the last injection to the FD” was unable to significantly predict the degree of standardized strength (**C**) and standardized thickness (**D**) asymmetry in dystonic musicians. The four patients who were still under the effect of BoNT in plots (**C**,**D**) are presented only for descriptive purposes and are not included in the regression analysis. For these four patients, the average standardized thickness asymmetry is −14.7 ± 14.1 (M ± SD), the standardized strength flexion is −71 ± 18.4 (M ± SD), and the standardized strength extension is −0.6 ± 18.9 (M ± SD). Abbreviations: BoNT = Botulinum toxin; FD = flexor digitorum superficialis and profundus.

BoNT-treatment-related parameters (i.e., “total amount of BoNT to the FD”, “total number of injections to the FD”, “averaged time between injections to the FD”, “total treatment time of the FD”, and “averaged dosage per injection to the FD”; FD = flexor digitorum superficialis and profundus) were included in a regression analysis in order to explain the remaining strength and thickness reduction presented after the termination of the treatment. The results indicated that only the “total amount of BoNT to the FD” injected during the entire treatment period was able to significantly predict the degree of weakness and atrophy. In particular, the higher the total amount of BoNT, the larger the asymmetry towards the non-injected hand for thickness and strength, meaning the higher the degree of weakness and atrophy in the injected hand, respectively. None of the other BoNT-treatment-related parameters were able to significantly predict the thickness or strength of the FD. Significant results from the regression analyses are presented in Table 1 and Figure 2A,B.

To estimate whether any strength and/or thickness recovery could be achieved after the termination of the BoNT treatment, we explored whether both variables could be predicted by the “time after the last FD injection”. Besides the slight positive slopes, the regression results were not significant. For descriptive and comparative purposes, data were plotted in Figure 2C,D together with the four patients who were still under BoNT effects.

Finally, no correlations between the unstandardized flexor thickness, strength flexion, and strength extension were found for dystonic or healthy musicians, apart from a correlation between thickness and strength in the dystonic group (*r* = 0.610, *p* < 0.05). Correlations between standardized flexor thickness, strength flexion, and strength extension and the various BoNT-treatment-related parameters among dystonic musicians are presented in Table 2.

## 3. Discussion

The results of the current study revealed that BoNT injections in the flexor digitorum superficialis (FDS) and flexor digitorum profundus (FDP) led to long-lasting atrophy and weakness of the injected muscles. The amount of time since the last injection was unable to predict any significant recovery effects. However, the results indicated that the degree to which atrophy and weakness occurred depended primarily on the overall amount of BoNT administered throughout the entire treatment period.

While strength loss is a well-documented side effect of BoNT injection therapy, atrophy has not been studied to such an extent (for a review, see [25]). Assessments concerning muscular atrophy after BoNT injections have mainly been conducted in animal model studies [17,18,20,21,22], whereas studies on human subjects are rather rare [16]. In our study, an overall flexion strength asymmetry of −9% towards the non-dominant hand was detected in the patient group even after the termination of BoNT treatment. In comparison to the healthy controls (who had an average +3.5% strength asymmetry towards the dominant hand), this finding indicates a strength loss of the injected FDS and FDP of about 12.5% (see Figure 1). Likewise, a non-dominant hand superiority concerning the muscle mass of the FDS and FDP muscle was expressed through a hand asymmetry of −6% in the patient group. As compared to the healthy matched controls, who had a dominant hand superiority of +5%, this finding indicates a muscular atrophy of the injected FDS and FDP of about 11%. With respect to the strength of the extensor digitorum (antagonist muscle), which was assessed as an additional control measurement, no differences between the two groups were observed. This might be an indication that the observed long-term weakness in the flexor digitorum muscle caused by a number of BoNT injections did not affect the strength of its antagonist (i.e., the extensor digitorum). However, results deriving from the strength assessment of the extensor digitorum should be interpreted with caution. Besides its excellent intraclass correlation coefficient (>0.92), reported by Ioannou et al. [24], strength assessments of the extensor digitorum showed a larger detectable change (≤12%) as compared to the detectable change of the thickness and strength of the flexor digitorum (≤5.1%). This may also explain the larger variability of data in this particular assessment (see Figure 1).

The regression analysis indicated that the amount of atrophy and weakness was significantly predicted (with about 39% of explained variance for each) by the overall amount of BoNT received during the entire treatment period. The effect of the total amount of BoNT has also been demonstrated in previous studies [20,21,26]. For instance, in a rat model study by Frick et al. [20], atrophy as well as weakness showed significant dose dependency after a single injection of BoNT. The onset of recovery was found to be dose-dependent as well [21], and a higher risk for the development of atrophy with later recovery onsets occurred for higher doses. Furthermore, a late onset of recovery allowed changes to occur in the contractile material within each muscle. It may lead to fibrosis, an increase in fatty tissue, and an overall loss of contractile fibers, resulting in a lower muscle quality with incomplete recovery of functional properties [16,18,20,21,27].

While the overall amount of BoNT units could predict the development of atrophy and weakness, the total number of BoNT injections, the total treatment period, and the inter-injection intervals did not. Based on these results and the fact that there was still another ~61% of unexplained variance, we assume that the total treatment time and the number of injections (including time interval between them) may not put the patient at a higher risk of developing atrophy and weakness. Concerning the amount of BoNT, which was the significant predictor of our regression model, an amount of up to 100 BoNT units in total could still cause the detectable long-term atrophy of −6% ± 0.7% [SE] (about 11% less than the average control group) after the termination of the treatment. Up to 300 units and 500 units of BoNT administered into the FDS and FDP in total could cause an atrophy of up to −11% ± 3.8% (SE) and −16 ± 8.4% (SE), respectively (about 16% and 21% less than the average control group value, respectively). For these numbers (100, 300, 500) of BoNT units, a weakness up to −7% ± 1%, −13 ± 5.4%, and −20% ± 11.8%, respectively, could be detected in the patient group (about 11%, 17%, and 24% less strength than the average control group).

Keeping the BoNT treatment dose low may still lead to satisfying results. This was, for example, shown in patients with cervical dystonia, facial hemispasm, and blepharospasm [28]. The Dysport doses administered were roughly half of the recommended dose for each injection site. Patient satisfaction was good under this regime while significantly fewer side effects such as dysphagia or ptosis occurred. In musicians suffering from focal hand dystonia, the administered BoNT doses, which are chosen depending on the severity of the symptoms and the estimated muscular strength of each patient, aim to eliminate symptoms such as cramps or curls of the fingers, allowing musicians to execute the fine-motor movements required to play musical instruments. The perception of patients’ motor skills and abilities to execute their musical instrument was subjectively estimated in the present study (see Table 3). The cohort that discontinued their BoNT therapy showed very similar percentages of motor skill between the time they were diagnosed (56% ± 31%) and the time of the experiment (58% ± 30%), indicating a stabilization of symptoms. The treatment of a musician’s dystonia is a highly individual decision, where achieving the stabilization of symptoms can in some cases be the preferred approach over improving symptom control at the cost of side effects. The fact that the motor ability was subjectively improved by about 20% in the four patients who were still under the effect of BoNT could emphasize the well-known effectiveness of the treatment already reported in several studies. At the same time, these patients seemed to present larger weakness (see Figure 2C,D). However, in order to draw safe conclusions and make comparisons between patients who terminated their BoNT treatment and those still under active treatment, a larger group of patients who are still under the BoNT effect is needed. Decreasing the BoNT dose to a lower degree that still satisfies dystonic musicians might decrease not only the long-term side effects but may decrease the short-term side effects as well: a hypothesis which could be further explored in future studies.

Finally, the amount of atrophy and weakness was unable to be predicted by the time since the last BoNT injection (averaged: 18 ± 12 months, min: 6, max: 41 months), indicating no significant recovery over a time frame of up to three and a half years after the last treatment session. While the desired effect of decreased over-contractions of the affected muscles wears off and makes re-injections necessary at an average interval of 3–4 months [5], the side effects of strength loss and atrophy persist significantly longer, at least in patients who receive multiple injections. Long-lasting side effects of BoNT injections were also reported by Schroeder et al. [16], who investigated the BoNT effect in a double-blind placebo-controlled study on two human test subjects. In this study, a neurogenic atrophy was still detectable in a muscle biopsy 12 months after a single BoNT injection. Side effect duration was also examined in animal model studies that found neurogenic atrophy still present at the end of the respective observation periods of six months [18] and one year [22]. Although muscle quality was not an outcome of our study, an increased echogenicity (i.e., an indirect parameter of muscle quality [30,31]) was observed in several patients during the ultrasound examination, suggesting the replacement of muscular tissue by fatty or fibrous tissue. Although our findings suggest that there is no tendency of a side effect recovery within a time frame of up to three years after the cessation of treatment, muscle quality would be worth examining in patients who interrupted their BoNT therapies up to 10 years ago. Furthermore, it is still unclear whether long-term side effects of BoNT in humans are at least in part caused by retrograde axonal transport to the spinal cord and alterations of spinal motor neurons as demonstrated in animal experiments (e.g., [32]). Here, future detailed electrophysiological studies, including an assessment of fibrillations in the respective muscles and recording of f-waves might clarify this hypothesis.

When considering the four patients who were still under the effect of BoNT (see Figure 2C,D), the occurrence of muscular strength loss and mass showed different dynamics. Although only four patients were examined to explore this effect, their strength loss appears to be almost thrice as severe as the development of atrophy and strength recovers at a much faster rate than muscle mass once the initial effect of BoNT passes. This is in line with the findings of Ma et al. [22], who performed electrophysiological measurements on rats that received BoNT injections. They found that muscle force generation decreased by 88% compared to the control side one week after injection, while muscle mass was reduced by only 32% after two weeks. In a study on New Zealand White rabbits by Fortuna et al. [19], an average atrophy of 53% was found one month after repeated injections of BoNT. Strength, however, was reduced by over 90% after the same interval. That strength loss can exceed the level of atrophy has also been shown in a study unrelated to BoNT treatments by Hortobágyi et al. [33]. After three weeks of leg immobilization in 42 human subjects, the reported average strength loss was 47%, while atrophy was only 11%. This discrepancy in dynamics between the recovery of strength and atrophy seemed to be similar during the short-term effect of BoNT (about 3 months) and even after that time. This was confirmed by Ma et al. [22] in a rat model study where force generation recovered by 65% within three months and muscle mass only recovered by 8%. Only after one year did both parameters recover to 95% and 96% of the control side values, respectively. Additionally, it has been shown that treadmill training after BoNT injections leads to an increase in strength but not muscle mass (for a review, see [34]). That strength can be gained without a major increase in muscle size was shown using MRI cross-section images of healthy participants before and after a strength training protocol [35,36]. A possible explanation could be an adaptation of the nervous system that allows greater force generation without any morphological changes in the muscle itself [24,37,38].

Exploring the side effects of BoNT injections in focal dystonia patients remains challenging. A more precise evaluation could be based on measurements of strength (and atrophy) before and after each injection. However, since the effect of BoNT injections takes 2–5 days to fully develop [25], patients would have to come back to the clinic after this time period for a meaningful post-injection measurement, rendering this approach impractical. Another factor that contributes to a higher variability among dystonic musicians is the inter-injection interval. In our study, the average time between injections ranged from ~1.8 months to ~21 months. A similar inter-injection range for dystonic musicians was reported by Jabusch et al. [39]. The criteria for the next injection among instrumental musicians affected by upper limb dystonia are often based on upcoming scheduled concerts or competitions for which they need to have maximum control of the fingers. In patients with other forms of focal dystonia, for instance, cervical dystonia, where the symptoms are more related to impairment in daily activities and pain, inter-injection intervals are shorter (~108 days [40,41]); the time interval is associated primarily with the duration of BoNT effects (which start to wear off after 2–3 months) [26].

By observing the distribution of all cases carefully, it is becoming obvious that the long-lasting side effects of BoNT injections differ from patient to patient and could depend on further physiological and individual factors (e.g., individual regeneration potential, muscle quality, muscular strength training between injections, etc.). In particular, the investigation of the muscular tissue quality after BoNT injections using ultrasonography deserves future examination. Echogenicity captured by ultrasonography serves as an indirect parameter of muscle quality since fatty and fibrous tissues result in a higher echogenicity [30,31].

Studies on BoNT effects conducted in humans via the usage of objective assessments remain rare and challenging to achieve. However, future studies should target a higher sample size, including patients who interrupted their BoNT treatment for longer than 3.5 years. Moreover, since the time interval between injections varies a lot among dystonic musicians, similar studies in patients with other forms of focal dystonia, for instance, cervical dystonia [40,41], should also be conducted. Examining patients with smaller and more regular inter-injection intervals, which in turn affect the total BoNT treatment time, could provide a further understanding of the remaining 61% unexplained variance in our regression analysis.

## 4. Conclusions

The current study showed that multiple BoNT injections in the FDS and FDP muscle in patients suffering from focal hand dystonia can lead to 11% and 13% of atrophy and weakness, respectively, even up to three and a half years after the termination of treatment. Both side effects depend primarily on the total amount of BoNT received during the overall treatment period. In contrast, the number of injections and their distribution throughout the overall treatment time do not seem to play a significant role. Nevertheless, the observed long-term side effects of BoNT remain subtle, emphasizing its safety over longer periods of time [10,11]. Finally, BoNT doses should be as high as necessary, but more importantly, low enough to avoid long-term side effects.

## 5. Methods

### 5.1. Participants

Twelve right-handed [29] musicians affected by focal hand dystonia in their dominant (right) hand who had received BoNT treatment in the past comprised the main experimental group of the current study. All of them were responders to the BoNT injection therapy. However, after several years of repeated BoNT injections, they decided to terminate their injection therapy for various reasons [15,23]. Their last BoNT injection was received within a time frame of 165 to 1239 days (5.5–41.3 months) before the day of the experiment. All patients had received BoNT injections in the superficial and/or profound flexor digitorum muscle (FDS and FDP). The most frequently injected finger fascicles of the FDS and FDP were the digits II, III, and IV. Moreover, over the course of the BoNT injection therapy, four patients received additional BoNT injections in some adjacent muscles (the M. flexor and extensor carpi radialis, the M. interosseus palmaris, and the muscles of the thumb), with two of them having received some of those injections (4 and 2 injections, respectively) after their last injection in the FDS and FDP. The total BoNT units injected in the FDS and FDP of the whole patient group represented 86.4% of the overall number of BoNT units applied. The remaining 13.6% were injected in the adjacent muscles mentioned above. All assessments of the current study focused strictly on the FDS and FDP muscle. Since most patients received Xeomin (Incobotulinumtoxin A), all BoNT doses were standardized in the Xeomin equivalent based on the ratio Xeomin:Botox (OnabotulinumtoxinA) 1:1 and Xeomin:Dysport (Abobotulinumtoxin A) 1:3 [42], meaning that 1 unit of Xeomin was equivalent to 1 unit of Botox and 3 units of Dysport, respectively. For further information concerning demographics and BoNT-related information, see Table 3.

Finally, four additional patients who were under active BoNT treatment (meaning they received their last injection in the FDS and FDP within the last four months before the experimental procedure [8,9]) were also recruited. Data from these four patients (and their respective matched controls) were used only for descriptive purposes. All patients were recruited from the database of the outpatient Clinic/Institute of Music Physiology and Musicians’ Medicine in Hanover, Germany.

The control group consisted of 12 healthy musicians (plus 4 healthy matched controls for the patients who were currently under the BoNT effect), all matched for handedness, instrument, gender, and as closely as possible for age [24]. Exclusion criteria for both groups were possible orthopedic or other neurological disorders (e.g., surgery, nerve damage, recent broken bones, neuromuscular disorders, etc.) known to affect the upper limbs, specifically the strength and muscle mass (thickness). Further participants’ characteristics are presented in Table 3. All participants gave their informed consent prior to participation and the study was approved by the ethics committee of the Hanover Medical School.

### 5.2. Assessments

**Figure 3:**
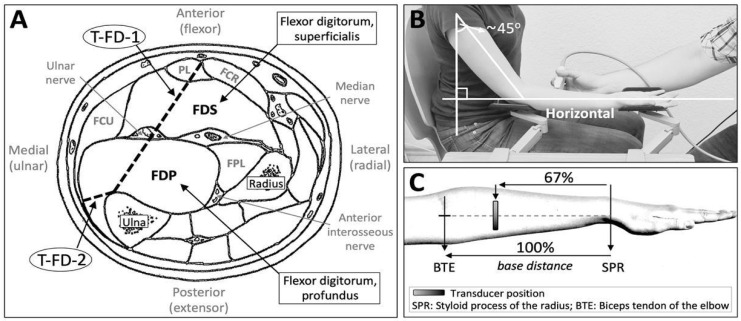
(**A**) Ultrasound assessments of the muscular thickness of the flexor digitorum muscles (superficialis and profundus). Abbreviations: T-FD-1 = thickness assessment of the FD, 1st measurement; T-FD-2 = thickness assessment of the FD, 2nd measurement; FCR = flexor carpi radialis; FCU = flexor carpi ulnaris; FPL = flexor pollicis longus; PL = palmaris longus (the template of Figure (**A**) was received and modified with permission from Sklerov and Pullman [45] and the original source [46], Charles C Thomas Publisher, Ltd., Springfield, IL, USA). (**B**) Position of the upper limb during ultrasound assessment. (**C**) Hand landmarks for the standardization of the ultrasound measurement position. (Figures (**B**,**C**) from [44] with permission from the authors and publisher; © 2019 Hodde et al. Muscle & Nerve published by Wiley Periodicals Inc., Hoboken, NJ, USA).

*Ultrasound assessment*: Since the superficial and profound parts of the M. flexor digitorum are similarly involved in the flexion of the index to the little finger (II–V) [43,44], and due to the fact that in all except one of our patients (n = 15 across two patient groups, 94%) injections were applied in fascicles of both the FDS and FDP, the two compartments were treated as one unit and named flexor digitorum (FD) for the purpose of the measurement [24]. As a parameter for muscular atrophy, the thickness of the FD was measured via ultrasonography. Specifically, the thickness was estimated by measuring the following two distances of the FD: (a) between the ulnar side of the flexor carpi radialis muscle and the margo anterior of the ulna bone, and (b) between the ulnar side of the flexor carpi ulnaris muscle and the margo anterior of the ulna bone (Figure 3A). Both distances were averaged to serve as the averaged thickness of the FD. Imaging was performed in a well-standardized position in each upper limb while participants were sitting in an upright position (Figure 3B), [24]. Images were captured (B-mode) with a linear transducer (SL1543, 3.0–13.0 MHz) connected to a MyLab Six Ultrasound System (Esaote, Maastricht, Netherlands). The transducer was placed perpendicularly to the longitudinal axis of the muscle of interest and was coated with water-based gel, while pressure was reduced to the minimum level to prevent compression of the muscle during imaging (Figure 3C).

*Muscular strength*: As a parameter for muscular weakness, individual finger strength was measured in both hands in flexion and extension using a custom-made device available at the clinic. The device assesses the flexion and extension strength of each individual finger (II–V), while the palm, the wrist, and the lower arm are attached to it horizontally. All individual flexion and extension measurements, respectively, were averaged together resulting in one strength-flexion and strength-extension value for each hand. Extension strength was assessed to serve as a control parameter.

The above thickness and strength assessments have previously indicated excellent test-retest reliability, reproducibility, and accuracy [24]. Finally, the Symmetry Index between the right and left hands was calculated for both the thickness (flexor) and strength (flexion and extension) assessments and compared between groups (see the statistical analysis below).

### 5.3. Procedure

Each individual assessment was performed three times. The resulting averaged values of the three individual measurements were used for the statistical analysis [47,48]. The intra-rater reliability (i.e., degree of agreement among the three repeated measurements performed by a single examiner) indicated excellent reliability for the individual thickness measurements and high-to-excellent reliability for the individual flexion and extension strength assessments [24]. The order of the experimental sessions was the same for each participant: (a) ultrasound, (b) 1st strength assessment, (c) 1st questionnaire session, (d) 2nd strength assessment, (e) 2nd questionnaire session, and (f) 3rd strength assessment. In order to avoid fatigue or training effects during the strength assessments, the order of the fingers (II–V) during flexion and extension, the order of the movement itself (flexion and extension), and the order of the hand (right/left) occurred in a randomized order. Participants were instructed to perform each strength assessment at their maximum power. To provide breaks between the three strength sessions, the questionnaire was divided into two parts. During the first questionnaire session, demographic and medical information was inquired. This also included a subjective self-rating of the personal motor skills on the instrument at the time of diagnosis and at the time of the experiment as a subjective measure for symptom control. The second questionnaire session included occupation-related information (main instrument, years of experience, practicing habits, etc.), as well as the Edinburgh Handedness Inventory [41].

### 5.4. Statistical Analysis

Asymmetry between hands for the above assessments was estimated with the Symmetry Index (SI) calculated as: $\left(\frac{X_{D}-X_{ND}}{0.5\times \left(X_{D}+X_{ND}\right)}\right)\times 100.$ Assuming that *X_D_* > *X_ND_*, where *X_D_* and *X_ND_* indicate assessments of the dominant (D) and the non-dominant hand (ND), respectively, SI estimates as a percentage the level of asymmetry between the two hands. Zero percent indicates no asymmetry, whereas positive or negative percentages indicate larger mass (thickness) or strength to the dominant or to the non-dominant side, respectively [49].

Inferential statistics using the asymmetry values were performed between the 12 dystonic musicians and the 12 healthy controls. The 4 patients who were still under the effect of BoNT and their 4 matched controls were used only for descriptive purposes. Independent t-tests were used for group comparisons, and Bonferroni corrections were applied to control for the inflation of a Type I error resulting from multiple comparisons. For the regression and correlation analysis, any thickness and strength asymmetry (X) of dystonic musicians (DM) caused by the different musical instruments’ demands (e.g., the extensive use of the left hand in violinists as compared to the right hand in pianists) were removed/standardized (X_DM_-X_HM_) according to the asymmetry values of their matched controls (HM) [50,51,52,53]. For the regression analysis, linear regression tests were used to predict the effect of variables of interest on standardized thickness and strength, whereas for associations between variables, the Pearson’s product moment correlation coefficient (or Spearman’s *r*) was used.

The assumptions of normality and homogeneity of variance were tested with Shapiro–Wilks and Levene’s tests, respectively, whereas linearity, homoscedasticity, and absence of multicollinearity were confirmed before regression. The level of significance was set at *p* < 0.05. Effect sizes were estimated with Pearson’s correlation coefficient, *r*. A statistical analysis was performed using IBM SPSS Statistics software for Macintosh (version 26). One extreme outlier value was identified in the variable “averaged time between injections”. This value was re-estimated (predicted) based on the values of the “strength flexion” variable as the *b*_0_ + *b*_1_ * “strength flexion” value, which resulted from a linear regression model (*F* (1,10) = 6.53, *p* < 0.05 with an *R*^2^ of 0.395).

## Figures and Tables

**Table 1:** Linear regressions (n = 12).

			Constant		IVs		
Variables		R^2^	B	SE B	B	SE B	β
**Outcome: Standardized Thickness Asymmetry of FD (%)**							
Total amount of BoNT (Units)		0.387	−4.088	3.063	−0.023	0.009	−0.622 *
**Outcome: Standardized Strength Asymmetry of FD (%)**							
Total amount of BoNT (Units)		0.391	−3.507	4.198	−0.032	0.013	−0.625 *

Thickness and strength asymmetry values (X) were standardized based on the asymmetry values of their matched controls (X_DM_-X_HM_) in order to eliminate any muscular effects caused by the different musical instruments’ demands (e.g., the extensive use of the left hand in violinists as compared to the right hand in pianists). * *p* < 0.05. Abbreviations: BoNT = Botulinum toxin; FD = flexor digitorum superficialis and profundus.

**Table 2:** Associations among the three standardized hand asymmetry variables (thickness and strength of the FD and strength of the extensor digitorum) and other BoNT treatment-related variables for the group of dystonic musicians (n = 12).

Variables		a.	b.	c.	d.	e.	f.	g.	h.
a.	Standardized Thickness Asymmetry of FD (%)								
b.	Standardized Strength Asymmetry of FD (%)	0.343							
c.	Standardized Strength Asymmetry of Ext. Digitorum (%)	0.308	0.422						
d.	Total amount of BoNT to the FD (units)	−0.678 *	−0.483	−0.294					
e.	Total Number of Injections to the FD	−0.449	−0.502	−0.651 *	0.710 *				
f.	Averaged time between injections to the FD (days)	0.112	0.341	0.014	−0.119	−0.096			
g.	Total treatment time from first to last BoNT injection to the FD (days)	−0.399	−0.28	−0.696 *	0.531	0.942 **	0.111		
h.	Time after last BoNT injection to the FD (until day of experiment) (days)	0.266	0.322	0.326	−0.622 *	−0.097	0.056	0.021	
i.	Averaged BoNT dosage per injection to the FD (units)	−0.14	−0.287	0.329	0.063	−0.465	0.102	−0.533	−0.414

Due to the violation of normality, correlations involving the “Standardized Thickness Asymmetry of FD”, and the “Total amount of BoNT” were performed with Spearman’s correlation coefficient. * Correlation is significant at the 0.05 level (2-tailed). ** Correlation is significant at the 0.01 level (2-tailed). Abbreviations: BoNT = Botulinum toxin; FD = flexor digitorum superficialis and profundus.

**Table 3:** Participants’ characteristics.

Characteristics	Dystonic Musicians (n = 12)	Healthy Musicians (n = 12)	Dystonic Musicians (under BTX, n = 4)	Healthy Musicians (n = 4)
Age in years (M±SD)	56.5 ± 8	57.4 ± 7	50 ± 6	51.5 ±2.5
Main instrument: piano/guitar/clarinet-oboe (n)	7/4/1	7/4/1	piano/guitar/trumpet/contrabass	piano/guitar/trumpet/contrabass
Handedness ^1^ (%)	90 ± 16	88 ± 17	93 ± 8	93 ± 9
Gender: Male / Female (n)	9 / 3	9 / 3	4 / 0	4 / 0
Dystonic symptoms onset (age, years: M ± SD)	39 ± 12	-	36 ± 6	
Motor skills after diagnosis ^2^ (out of 100%)	56 ± 31	-	52.5 ± 17	
Motor skills today ^2^ (out of 100%)	58 ± 30	-	70 ± 21	
Cumulative hours of practicing	33,413 ± 21,272	45,855 ± 26,075	41,769 ± 13,188	43,481 ± 19,584
Age started playing in years (M ± SD)	10.4 ± 3.1	9.7 ± 4.3	13 ± 5	9 ± 2
Years of experience (M ± SD)	46 ± 8	48 ± 7	37 ± 2	43 ± 3
Body Mass Index (kg/m^2^)	26.5 ± 3.8	29.2 ± 4.6	25 ± 4	25 ± 2
Total (sum) and averaged (M ± SD) amount of BoNT (units) to the FD	3357, 280 ± 180	-	2571, 642 ± 322	-
Total number of injections to the FD (M ± SD)	16.5 ± 11	-	27 ± 17	-
Total treatment time (days) from first to last BoNT injection to the FD (M ± SD)	2801 ± 1877	-	2588 ± 1532	-
Averaged time between injections to the FD (days, M ± SD)	173 ± 29	-	113 ± 51	-
Time (days) after last BoNT (units) injection to the FD (until day of experiment) (M ± SD)	531 ± 361	-	73 ± 30	-
Average BoNT dosage (units) per injection to the FD (M ± SD)	20 ± 10	-	26 ± 6	-

No significant differences (*p* > 0.05) were found between patients (n = 12) and healthy matched controls (n = 12) with respect to age, years of experience, age started playing, cumulative hours of practicing, body mass index, and handedness percentage. ^1^ (Handedness percentage is defined as: left-handed < −40%; right-handed > +40%; ambidextrous otherwise, [29]). ^2^ Motor skills concerning musical performance were self-reported subjective estimations completed by the patients themselves. Abbreviations: BoNT = Botulinum toxin; FD = flexor digitorum superficialis and profundus.

## Data Availability

Data are available upon request.
